# WWII traumatic events, subjective well-being and valuation of life in the very old

**DOI:** 10.1007/s00391-021-01906-7

**Published:** 2021-06-11

**Authors:** Daniel Hauber, Roman Kaspar, Susanne Zank

**Affiliations:** 1 Chair of Rehabilitative Gerontology, Department of Special Education and Rehabilitation Science, Faculty of Human Sciences, University of Cologne, Herbert-Lewin-Str. 2, 50931 Cologne, Germany; 2 Cologne Center for Ethics, Rights, Economics, and Social Sciences of Health, University of Cologne, Cologne, Germany

**Keywords:** Oldest old, War, Trauma, Depression, Resilience, Hochaltrige Personen, Krieg, Trauma, Depression, Resilienz

## Abstract

**Background:**

Experiencing war is a major trigger for physical and mental health problems. People in the German population who are currently over 80 years of age experienced the Second World War (WWII) as children or adolescents, at a time when psychological vulnerability is high. Empirical results show that positive subjective well-being (SWB) and valuation of life (VoL) in older cohorts are widespread; however, when confronted with existential age-associated changes, many older adults experience increased burden, sometimes bringing biographical vulnerabilities to the forefront. This study investigated SWB and VoL in the very old and examined the influence of negative WWII experiences on these outcomes.

**Method:**

Cross-sectional data from the “Survey on quality of life and subjective well-being of the very old in North Rhine-Westphalia (NRW80+)” are presented. Multiple regression models, adjusted for gender, age, physical health, and full inpatient care, were computed to assess the impact of suffering from the effects of WWII traumatic experiences on SWB and VoL.

**Results:**

Over 13% spontaneously reported suffering from the effects of WWII events and an additional 29% reported negative experiences when explicitly asked about them. Multiple regression models showed elevated depression scores for participants suffering from the effects of WWII traumatic events. No association with positive affect was found. Suffering from the effects of WWII traumatic events did not influence VoL engagement with life or VoL optimism.

**Discussion:**

Many very old adults still seem to struggle with the repercussions of WWII traumatic experiences. Future studies could further examine if the missing association with positive affect and VoL is a sign of resilience.

## Abstract (de)

**Hintergrund:**

Krieg zu erleben ist ein maßgeblicher Auslöser für körperliche und psychische Erkrankungen. Die hochaltrige deutsche Bevölkerung (> 80 Jahre) hat den Zweiten Weltkrieg (WK 2) im Kindes- oder Jugendalter und damit in einer Phase, in der die Psyche besonders verwundbar ist, erlebt. Empirische Studien zeigen einerseits, dass subjektives Wohlbefinden (SWB) und Wertschätzung des Lebens (VoL) in älteren Kohorten weit verbreitet sind. Andererseits erleben viele Menschen, die mit altersbedingten Belastungen konfrontiert sind, verstärkt negative Gedanken und Gefühle auch im Zusammenhang mit traumatischen Ereignissen aus Kindheit und Jugend. Unsere Studie untersucht SWB und VoL in der hochaltrigen Bevölkerung sowie den Einfluss traumatischer WK-2-Erlebnisse auf beide Aspekte.

**Methode:**

Es werden Querschnittsdaten aus der Studie „Lebensqualität und Wohlbefinden hochaltriger Menschen in NRW (NRW80+)“ vorgestellt. Es wurden multiple Regressionsmodelle berechnet, um die Auswirkung traumatischer WK-2-Erlebnisse auf SWB und VoL abzuschätzen. Die Modelle waren adjustiert für Geschlecht, Alter, körperliche Gesundheit und vollstationäre Pflege.

**Ergebnisse:**

Über 13 % der Befragten gaben spontan an, unter den Auswirkungen von WK-2-Ereignissen zu leiden, und weitere 29 % berichteten auf Nachfrage davon. Die Regressionsmodelle ergaben für Personen, die berichteten, unter den Auswirkungen von traumatischer WK-2-Ereignisse zu leiden, höhere Depressionswerte. Es wurde kein Zusammenhang mit positivem Affekt gefunden. Als belastend empfundene traumatische WK-2-Erlebnisse hatten keinen Einfluss auf VoL-Engagement und Optimismus.

**Diskussion:**

Ein großer Anteil der deutschen Hochaltrigen scheint immer noch mit der Bewältigung traumatischer WK-2-Erlebnisse befasst zu sein. Zukünftige Studien könnten näher untersuchen, ob die fehlende Verknüpfung mit positivem Affekt und VoL ein Zeichen von Resilienz ist.

## Background

Traumatic events are known to have harmful effects on health throughout life [3]. Around 70% of the global population experience at least one traumatic event throughout their lifetime [19]. Many old people intentionally or unintentionally re-engage with early life traumatic experiences, which is often perceived as stressful [4, 5]. Nevertheless, positive subjective well-being (SWB; i.e. positive reactions to one’s life [6]) and high valuation of life (VoL; affectpive and cognitive appraisals of the perceived value of one’s life [24]) seem to be widespread in the very old population [17, 33]. While the very old population (≥ 80 years) is expected to triple by the middle of this century [12] studies about the long-lasting effects of traumatic events on SWB and VoL in this specific population are still rare.

In Germany, scholars with a background in psychodynamic psychotherapy pioneered work in the field of late effects of traumatic World War II (WWII) experiences, based on their clinical experiences [22, 26, 28, 29]. This was followed by epidemiological studies. In a representative study conducted in 2008, around 57% of all Germans aged 60–85 years said they had experienced at least 1 war-related traumatic event [10]. This group experienced WWII as children or adolescents, at a time when the vulnerability for stress-related disorders is elevated [3, 30]. Previous studies focused on the long-lasting pathological consequences of WWII-related traumatic events, such as posttraumatic stress disorder (PTSD). Selected subsamples showed a prevalence of PTSD in older adults between 1% in former WWII child soldiers [21] and 42% in former displaced children [35]. Compared to other traumatic WWII experiences, the experience of sexual violence was particularly associated with PTSD symptoms in older age [20]. War-related experiences that cannot be described as traumatic in the strict sense, such as the absence of the father in childhood, are also associated with psychiatric symptoms in older age [7]. A representative survey held in 2005 showed a PTSD prevalence of over 3% in older German adults born in 1945 or earlier (and over 7% when partial PTSD was included) [9]. The study by Glaesmer et al. [10] showed a similar prevalence of 4%. In a 25-year longitudinal study of 50 older adults born between 1933 and 1945, Hiltl et al. [16] found associations between childhood burden and psychosomatic disorders but not between WWII traumatic events and psychosomatic disorders in later life.

The long-term effects of traumatic events, lasting for years to decades, are complex and difficult to measure but many individuals experience more war-related thoughts, memories, and feelings after being confronted with the normative process of aging (e.g. retirement, loss of close relatives). In summary, there is ample evidence of the pathological effects of early WWII-related trauma; however, findings on nonpathological outcomes, such as SWB and VoL, are still insufficient. The present study sought to broaden the understanding of long-term effects of WWII traumatic experiences in the very old population by examining their associations with SWB and VoL.

## Method

### Subjects

This study used data from the “Survey on quality of life and subjective well-being of the very old in North Rhine-Westphalia (NRW80+)”, a representative survey of persons aged 80 years and above [38]. The youngest participants were born in July 1936. Sampling followed a 2-stage approach using communities as primary sampling units and individuals as secondary sampling units. Men and older persons were oversampled compared to census data in order to ensure sufficient participation of these groups. The data were therefore weighted for sampling design and study nonresponse and can be generalized to the very old population. A total of 8040 persons were contacted between August 2017 and February 2018 with a total of 1863 interviews being realized, including 176 proxy interviews.

### Instruments

#### WWII traumatic events

Participants were asked: “were there any experiences or events in your life that still weigh on you today?”. If so, the participants were asked: “which event still weighs on you the most today?”. The most burdening event was then classified as WWII-related or non-WWII-related. If the participants reported no current burden or a non-WWII-related event, they were asked specifically for WWII-events that cause ongoing suffering. The WWII events were categorized by the interviewer according to the categories of a modified version of the diagnostic expert system for mental disorders (Diagnostisches Expertensystem für psychische Störungen, Dia-X) trauma list [36, 37]. Two new categories (i.e. “hunger” and “experiences and actions as a soldier”) emerged from coding open answers.

#### Subjective well-being

A 4-item short form of the depression in old age scale [13, 14] was used to assess depressive symptoms within the previous 2 weeks (i.e. negative affect). The scale consisted of items such as “do you feel low?”. (0 = “no”, 1 = “yes”). Hence, the aggregated score ranged from 0 to 4. In the case of missing values, the sum score was retained if participants reported at least two symptoms.

Positive affect was assessed with a subscale of the short form of the positive and negative affect schedule [18]. Participants were asked how often they had felt “excited”, “enthusiastic”, “alert”, “inspired” and “determined” within the past 12 months. The answers were given on a 5-point Likert scale ranging from “never” (1) to “very often” (5).

#### Valuation of life

The VoL was measured with the positive valuation of life scale [24]. The 13 items (e.g. “do you feel hopeful right now?”) could be answered with “yes” (2), “neither/nor” (1) or “no” (0). Mean values were calculated and every participant received scores between 0 and 2 for the two subscales “engagement with life” and “optimism” [8].

#### Multimorbidity

The study used an extended version of the self-administered comorbidity questionnaire [31] to assess multimorbidity. Participants were presented a list of 19 medical conditions and were asked if they were currently being treated for the illnesses (1 = yes, 0 = no). Multimorbidity was calculated as the sum of positive answers, ranging from 0 to 19.

### Statistical analyses

Descriptive statistics were calculated with SPSS 25 (IBM Corp., Armonk, NY, USA). Differences in age, multimorbidity, SWB, and VoL between participants not suffering from the effects of WWII traumatic events and those who reported suffering were tested with independent samples *t*-tests. Differences in gender, share of proxy interviews, number of respondents in full inpatient care, family status, and education were tested with *χ*^*2*^-tests. The MPlus 8.4 [27] was used for multiple regression models to estimate the impact of suffering from the effects of WWII traumatic experiences on depressive symptoms, positive affect, VoL engagement with life, and VoL optimism. The regression models were adjusted for age, gender, multimorbidity, and full inpatient care. Standard errors of parameter estimates were corrected for the complex sampling design.

## Results

### Descriptive characteristics of the sample

The year of birth ranged between 1915 and 1937, with about 52% born between 1933 and 1937. Age at participation ranged from 80 to 102 years. Over 42% of respondents reported suffering from the effects of WWII traumatic events. Table 1 depicts the main descriptive characteristics of the sample. Those who reported suffering were older (Mean_years_ = 85.36, Standard Deviation_years_ = 4.21) than comparisons (*M*_years_ = 84.92, *SD*_years_ = 4.13). This difference was statistically significant (*t*(1823) = 2.21, *p* < 0.05), reflecting a small effect (Cohen’s *d* = 0.11). Participants suffering from the effects of WWII-related traumatic events were also treated for more medical conditions (*M*_medical conditions_ = 3.84, *SD*_medical conditions_ = 2.51) than the not affected group (*M*_medical conditions_ = 3.21, *SD*_medical conditions_ = 2.20). This difference was also statistically significant (*t*(1529.77) = 5.52, *p* < 0.001), reflecting a small effect (Cohen’s *d* = 0.27). The WWII-affected group was also better educated (*χ*^*2*^ = 8.14, *p* < 0.05) and less often in full inpatient care (9.23%) than comparisons (14.60%; *χ*^*2*^ = 11.59, *p* < 0.01). Subjects suffering from the effects of WWII traumatic events showed higher depression scores (*M*_depression score_ = 1.08, *SD*_depression score_ = 1.18) compared to the nonaffected group (*M*_depression score_ = 0.81, *SD*_depression score_ = 1.07). Again, this difference was statistically significant (*t*(1481.32) = 4.87, *p* < 0.001), reflecting a small effect (Cohen’s *d* = 0.24). There were no significant differences in positive affect, engagement, or optimism. No differences were found for gender, share of proxy interviews, and family status. Table 2 shows the most burdensome WWII events. Bombardment was the most burdensome event (30.37%), followed by displacement or flight (24.24%).

**Table 1:** Descriptive characteristics of the sample (*n* = 1863)^a^

	Not suffering from the effects of WWII traumatic event *n* = 1048 (57.45%)		Suffering from the effects of WWII traumatic event *n* = 777 (42.55%)		
		Missing values		Missing values	
	*M* (*SD*)/*n* (%)	(%)	*M* (*SD*)/*n* (%)	(%)	*t/χ*^*2*^
Age (years)	84.92 (4.13)	–	85.36 (4.21)	–	2.21*
*Female*	657 (62.68)	–	503 (64.74)	–	0.35
*Multimorbidity*	3.21 (2.20)	1.48	3.84 (2.51)	0.80	5.52***
*Full inpatient care*	153 (14.60)	0.15	72 (9.23)	0.26	11.59**
*Proxy interview*	85 (8.11)	–	62 (7.98)	–	0.01
*Family status*	–	–	–	–	8.18
Married/living together	418 (39.89)	–	267 (34.36)	–	–
Married/living separately	11 (1.05)	–	5 (0.64)	–	–
Divorced	45 (4.29)	–	33 (4.25)	–	–
Widowed	526 (50.19)	–	440 (56.63)	–	–
Single	48 (4.58)	–	33 (4.25)	–	–
*Education*	–	6.49	–	8.48	8.14*
Low	296 (28.24)	–	173 (22.27)	–	–
Middle	511 (48.76)	–	388 (49.94)	–	–
High	173 (16.51)	–	150 (19.31)	–	–
*DIA-S4 depression score*	0.81 (1.07)	7.57	1.08 (1.18)	6.43	4.87***
*PANAS positive affect*	3.23 (0.88)	3.39	3.30 (0.90)	1.96	1.69
*VoL optimism*	1.58 (0.42)	1.10	1.58 (0.42)	0.77	0.19
*VoL engagement with life*	1.46 (0.61)	1.99	1.45 (0.62)	2.56	0.30

Weighted data

*DIA-S4* Depression in old Age Scale, *PANAS* Positive and Negative Affect Schedule, *VoL* Valuation of Life, *M* mean, *SD* standard deviation

*p < 0.05, ** p < 0.01, *** p < 0.001

^a^*n* = 38 could not be assigned to either of the two groups

**Table 2:** Most burdensome WWII event (*n* = 777)

	*n* (%)
Bombardment	236 (30.37)
Displacement or flight	188 (24.20)
Other event	83 (10.68)
Physical threat, attack, injury, or torture	69 (8.88)
Death of parents	71 (9.14)
Death of siblings	47 (6.05)
Captivity, kidnapping, or hostage taking	29 (3.73)
Death of partner	13 (1.67)
Hunger	11 (1.42)
Experiences and actions as a soldier	10 (1.29)
Survived serious illness	5 (0.64)
Death of child	4 (0.51)
Rape	3 (0.39)

Weighted data

### WWII traumatic events as predictors of later life SWB and VoL

In a second step, four regression models assessed the relationship of suffering from the effects of WWII traumatic events with depression scores, positive affect, VoL engagement with life, and VoL optimism (Table 3). All analyses were controlled for age, gender, full inpatient care, and multimorbidity as possible confounders. All four models explained a significant amount of variance in the dependent variables (*R*^2^_depressive symptoms_ = 0.12, *p* < 0.001; *R*^2^_positive affect_ = 0.07, *p* < 0.001, *R*^2^_VoL engagement_ = 0.17, *p* < 0.001, *R*^2^_VoL optimism_ = 0.13, *p* < 0.001); however, suffering from the effects of WWII-related traumatic events was only significantly associated with depressive symptoms (*β* = 0.09, SE = 0.03, *p* < 0.01). Participants who reported suffering from the effects of a WWII traumatic event had significantly higher depression scores compared to participants who reported not suffering from the effects of WWII traumatic events.

**Table 3:** Multiple regression analyses assessing the relationship of suffering from the effects of WWII traumatic events with subjective well-being and valuation of life (*n* = 1825)

	Depressive symptoms	Positive affect	VoL engagement with life	VoL optimism
	*β *(SE)	*β *(SE)	*β *(SE)	*β *(SE)
Constant	−0.81 (0.50)	5.59 (0.55)***	4.85 (0.62)***	6.41 (0.54)***
Age	0.05 (0.02)*	−0.09 (0.03)**	−0.10 (0.03)**	−0.11 (0.03)***
Female	0.03 (0.02)	0.04 (0.03)	−0.05 (0.02)*	−0.03 (0.02)
Full inpatient care	0.15 (0.04)***	−0.20 (0.03)***	−0.34 (0.04)***	−0.28 (0.03)***
Multimorbidity	0.28 (0.03)***	−0.13 (0.03)***	−0.14 (0.03)***	−0.15 (0.03)***
Suffering from the effects of WWII traumatic event	0.09 (0.03)**	0.05 (0.03)	−0.01 (0.03)	0.00 (0.03)
R^2^	0.12***	0.07***	0.17***	0.13***

*VoL* Valuation of Life, *β* standardized regression coefficient, *SE* standard error

Weighted data

*p < 0.05, **p < 0.01, ***p < 0.001

## Discussion

This study examined WWII traumatic experiences that are perceived as burdening by today’s very old (≥80 years) and their association with SWB and VoL. To this end, a representative sample was used from Germany’s most populated federal state NRW. About 42% of the very old respondents stated that they perceive traumatic experiences related to WWII as stressful. Bombing and flight were most frequently mentioned as the most stressful experiences.

While most other studies aimed at the psychopathological consequences of WWII traumatic events (i.e. mainly PTSD), the present study focused on the effects on SWB and VoL. As expected, suffering from the effects of WWII traumatic events was associated with more negative affect (i.e. depressive symptoms), even after controlling for a wide range of vulnerabilities for depression in very old age (e.g. age, gender, number of treated medical conditions, and full inpatient care).

The results are in line with previous studies about traumatic WWII experiences in the very old German population. For example, 10 years earlier, Glaesmer et al. found that 57% of the population of older adults reported having experienced at least one traumatic war event, which was associated with higher risks for current depressive symptoms [10]. Experiencing civil and war traumatic events was also repeatedly associated with depressive symptoms in international studies (e.g. [2]). Considered together, this underlines the association of WWII traumatic events with negative affect in today’s very old population.

In the present study, suffering from WWII traumatic experiences was, however, not associated with positive affect. This finding supports the dual channel model of Lawton et al. [23], which postulates that positive affect is more closely related to outer influences, such as quality of time and friends, while negative affect is more closely related to inner influences, such as health.

Additionally, suffering from the effects of WWII traumatic events was not associated with VoL. The VoL can be divided into two distinct dimensions: optimism and engagement. Optimism is “a cognitive, optimistic and future-oriented stance towards life” [8], while engagement describes a state of “problem-solving, activation towards and confidence in obtaining one’s desired goals” [8]. The present results partly contradict the finding that traumatic war events are associated with lower satisfaction with life and quality of life in younger civilian [1] and veteran [32] populations.

The Later-Adulthood Trauma Reengagement (LATR) concept of Davison et al. [4, 5] provides one theoretical framework that helps to explain late life influences of early life traumatic war experiences. The authors found that many aging veterans who initially did not develop posttraumatic stress symptoms after traumatic experiences in war re-engaged with their traumatic experiences when confronted with their own aging. Heuft’s concept of trauma reactivation [15] focussed on processes that are not limited to veterans. He formulated that older individuals may deal with unresolved trauma due to more time resources (e.g. retirement) and often feel pressure to complete their unfinished tasks. In addition, experiencing helplessness due to physical aging can revive previous trauma. The results of the present study contribute in several ways to the understanding of these processes in the very old population. Firstly, more than 42% of the sample perceived WWII traumatic experiences, such as bombing or flight as burdening and, thus, might be in the process of trauma re-engagement. In addition, trauma re-engagement was associated with more negative affect; however, positive affect and VoL were not significantly affected by traumatic WWII experiences, adding to the previous body of research. Thus, the influence of traumatic WWII experiences appears to affect primarily negative mood but does not significantly affect other domains of psychological well-being. This finding should be further investigated in subsequent studies.

Nevertheless, the findings of the present study also have an encouraging component. As Glaesmer et al. [10] found in 2008, about 57% of the German older adults (60–85 years) reported having experienced traumatic WWII events. In the present study, a lower proportion of about 42% of the very old reported suffering from the effects of WWII traumatic experiences. Instead of interpreting the elevated negative affect as a negative outcome of the re-engagement with WWII traumatic events, the missing differences in positive affect, optimism, and engagement with life could be seen as a sign of resilience. Whereby resilience can be defined as a “process of adapting well in the face of adversity, trauma, tragedy, threats or even significant sources of stress” [34] and therefore should not be confused with the absence of symptoms. Future studies could implement measurements of posttraumatic growth (i.e. subjective experience of positive change after traumatic event) to further investigate this process in the very old population. At the same time, a possible cohort effect must be kept in mind when comparing the results of the present study with those of Glaesmer et al. [10].

The well-known limitations of cross-sectional designs regarding causality must be kept in mind when interpreting the present results. Depressed adults are known to be biased towards negative cognitions and memories [11] and could, thus, describe their own past more negatively compared to non-depressed adults. Longitudinal designs are needed in order to provide further insights. The present study used proxies if the target was unable to conduct the interview. Some studies indicate that proxies tend to overestimate the psychological burden of older adults (e.g. [25]). Nevertheless, information given by long-term partners, children or close affiliates may contribute to a more representative picture of the very old population.

## Conclusion

- About 42% of the very old German population feel burdened by traumatic events related to WWII.
- Suffering from the effects of WWII traumatic events is associated with negative affect.
- Practitioners should become familiar with the historical circumstances under which very old patients grew up and should be encouraged to screen for possible war traumatization.
- Behavior, such as aggressiveness or restlessness could be symptoms of (re)traumatization.
